# Growth Differentiation Factor 15 (GDF-15): A Novel Biomarker Associated with Poorer Respiratory Function in COVID-19

**DOI:** 10.3390/diagnostics11111998

**Published:** 2021-10-27

**Authors:** Leticia Alserawan, Patricia Peñacoba, Sandra Elizabet Orozco Echevarría, Diego Castillo, Esther Ortiz, Laura Martínez-Martínez, Esther Moga Naranjo, Pere Domingo, Ivan Castellví, Cándido Juárez, Anaís Mariscal

**Affiliations:** 1 Department of Immunology, Hospital de la Santa Creu i Sant Pau, Biomedical Research Institute Sant Pau (IIB Sant Pau), 08041 Barcelona, Spain; lalserawan@santpau.cat (L.A.); eortiz@santpau.cat (E.O.); lmartinezma@santpau.cat (L.M.-M.); mmoga@santpau.cat (E.M.N.); cjuarez@santpau.cat (C.J.); 2 Department of Respiratory Medicine, Hospital de la Santa Creu i Sant Pau, 08041 Barcelona, Spain; ppenacoba@santpau.cat (P.P.); sorozco@santpau.cat (S.E.O.E.); dcastillo@santpau.cat (D.C.); 3 Department of Internal Medicine, Hospital de la Santa Creu i Sant Pau, 08041 Barcelona, Spain; pdomingo@santpau.cat; 4 Department of Rheumatology, Hospital de la Santa Creu i Sant Pau, 08041 Barcelona, Spain; icastellvi@santpau.cat

**Keywords:** GDF-15, COVID-19, respiratory function, inflammation, biomarker

## Abstract

It is essential to find new biomarkers for severity stratification of patients with coronavirus disease (COVID-19). Growth differentiation factor 15 (GDF-15) is upregulated in pathological conditions that involve inflammation and/or oxidative stress. We determined circulating levels of GDF-15 and correlated them with clinical and laboratory parameters reflecting severity in 84 patients with COVID-19, finding that GDF-15 levels were higher in both patients than in 20 healthy controls and were higher in patients with poorer respiratory function. GDF-15 levels also correlated with interleukin-6, C-reactive protein, ferritin and D-dimer levels and with neutrophilia and lymphopenia. Of all the analysed biomarkers, GDF-15 showed the best area under the receiver operating characteristics curve in identifying patients with poor respiratory function. In conclusion, our data support GDF-15 as a biomarker associated with pulmonary impairment in COVID-19 and so can potentially be useful in stratifying COVID-19 cases by severity.

## 1. Introduction

Coronavirus disease (COVID-19) is a highly infectious respiratory disease caused by the new severe acute respiratory syndrome coronavirus 2 (SARS-CoV-2). While COVID-19 remains asymptomatic in some people, in others it is associated with severe complications, such as interstitial pneumonia and respiratory failure [1]. The great diversity in disease severity resulting from SARS-CoV-2 infection is partially explained by its interaction with the immune system. Hyperactivation of the immune response in some patients can lead to a cytokine storm, which has been associated with severe clinical manifestations of COVID-19 and poor therapeutic outcomes [1]. Innate immune cells such as macrophages acting as the first line of defence in these patients may produce interleukin-6 (IL-6), which, in turn, contributes to excessive inflammation. Serum levels of IL-6 and ferritin, as inflammatory biomarkers, are reported to be significantly increased in COVID-19 non-survivors compared to survivors [2], while patients with severe COVID-19 have been reported to have higher neutrophil and lower lymphocyte counts. Lymphocyte depletion and exhaustion in these patients may be a consequence of the overproduction of proinflammatory cytokines [1]. Other soluble factors are also increased in patients with COVID-19, including C-reactive protein (CRP), procalcitonin (PCT), lactate dehydrogenase (LDH), alanine aminotransaminase (ALT), aspartate transaminase (AST), troponin, D-dimer and fibrin degradation products [1,3]. D-dimer and fibrin degradation products, in particular, have been associated with COVID-19 mortality [4].

Due to COVID-19 becoming a worldwide pandemic, with high associated human and economic costs, the identification of new biomarkers to stratify patients according to the risk of poorer outcomes is crucial. One potential biomarker is growth differentiation factor 15 (GDF-15), also known as macrophage inhibitory cytokine (MIC-1), a member of the transforming growth factor-beta (TGF-β) superfamily that helps tissues survive inflammatory stress. While GDF-15 expression outside the reproductive organs is low to absent, it is upregulated in pathological conditions that involve inflammation and/or oxidative stress, e.g., cancer, cardiovascular disease, pulmonary disease, diabetes and renal disease [5]. In cardiovascular disease and pulmonary vascular disorders, elevated circulating levels of GDF-15 have been associated with mortality [6], while in acute respiratory distress syndrome (ARDS), GDF-15 has been associated with several secondary outcomes [7].

The role played by GDF-15 in COVID-19 is less well understood. Only a few studies have reported higher GDF-15 levels in COVID-19 patients than in healthy people [8,9]. GDF-15 levels have been associated with mortality and have been correlated with biomarkers such as IL-6, CRP, ferritin, D-dimer, serum calprotectin, PCT, troponin, pro b-type natriuretic peptide (proBNP) and viremia [9,10]. Our aim was to analyse GDF-15 levels in patients with COVID-19 and to correlate them with clinical and laboratory parameters of disease severity.

## 2. Materials and Methods

Our prospective study included 84 patients with COVID-19 admitted to the Hospital de la Santa Creu i Sant Pau (Barcelona, Spain) and 20 healthy controls. COVID-19 diagnosis was confirmed by a positive polymerase chain reaction (PCR) test.

On admission of patients with COVID-19, respiratory parameters such as oxygen saturation (SpO_2_) were measured using a pulse oximeter. In addition, to monitor the severity of acute hypoxic respiratory failure, the saturated oxygen (SpO_2_)/fraction of inspired oxygen (FiO_2_) ratio was calculated [11]. Blood samples were collected within a mean of 7 days (median 2 days) post-admission and the following analytical parameters were measured: GDF-15 (ELISA R&D Systems, Minneapolis, MN, USA), CRP (Immunoassay Alinity Analyser, Abbot Laboratories, Chicago, IL, USA), IL-6 (Elecsys IL-6 immunoassay Roche, Mannheim, Germany), D-dimer (Immunoassay ACL TOP Analyser, Werfen, Barcelona, Spain) and leukocyte and neutrophils counts (XN-10 Haematology Analyser, Sysmex, IL, USA). GDF-15 quantification was performed using ELISA kits from the same batch and in the same assay (the intra-assay variation coefficient was 2.8). The rest of the laboratory parameters were determined according to the clinical practice routine in our hospital.

The normal distribution of the data was tested using the Kolmogorov–Smirnov test. Normally distributed variables and non-parametrically distributed variables were reported as mean ± standard deviation (SD) and as median and interquartile range (IQR), respectively. Groups were compared using the student’s *t*-test, Mann–Whitney test or Wilcoxon test depending on the Gaussian distribution. Categorical variables were compared using Fisher’s test. Correlation analyses were carried out with Pearson’s or Spearman’s correlation depending on the Gaussian distribution. Area under the curve (AUC) of the receiver operating characteristic (ROC) curve was used to identify patients with poorer respiratory function (SpO_2_/FiO_2_ ≤ 400). Values with *p* < 0.05 were considered significant.

## 3. Results

### 3.1. Characteristics of Patients with COVID-19 on Admission

For the 84 patients hospitalised with COVID-19, the median (IQR) stay was 9 (6–14) days and mortality was 1.2%. On admission, median (IQR) SpO_2_ was 95% (94–97%) and median (IQR) SpO_2_/FiO_2_ was 395 (227.5–454.8). There were no differences in sex (*p* = 0.13) or age (*p* = 0.24) between patients with COVID-19 and healthy controls. Demographic, clinical and laboratory data for the 84 patients with COVID-19 are shown in Table 1.

### 3.2. Association between GDF-15 and Clinical Severity

**Figure 1:**
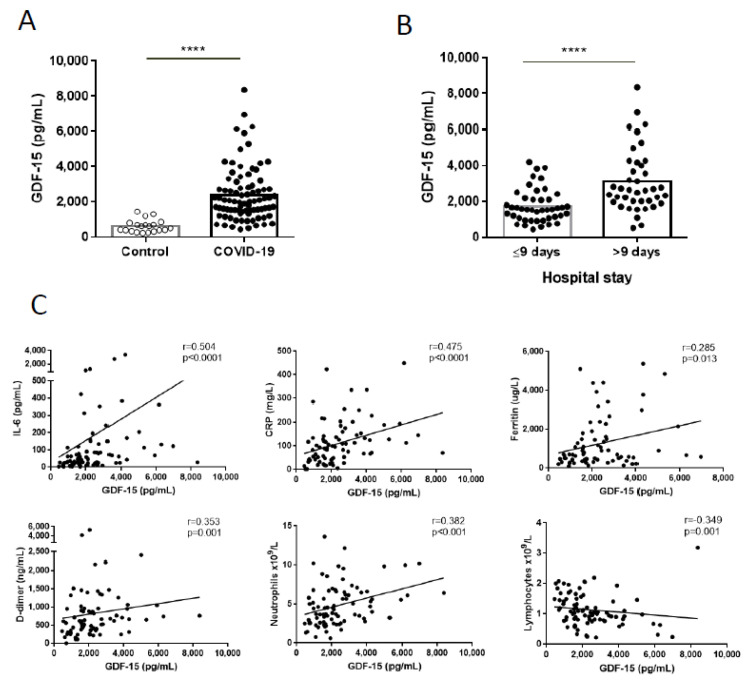
Serum growth differentiation factor 15 (GDF-15) levels. (**A**) GDF-15 levels in healthy controls (white circles) and in patients with COVID-19 (black circles); (**B**) GDF-15 levels in patients with COVID-19 by hospital stay; (**C**) GDF-15 correlations with other analytical markers. The Mann–Whitney test and Spearman’s correlation coefficient were used for statistical analysis. **** *p* < 0.0001.

Median (IQR) GDF-15 levels were higher in the patients with COVID-19 than in the healthy controls (Figure 1A): 2051 (1474–2925) pg/mL vs. 582 (370–807) pg/mL; *p* < 0.0001. No association between time of sampling and GDF-15 levels was observed (r = 0.051; *p* = 0.642). GDF-15 levels were also higher for those patients with a longer stay in the hospital (Figure 1B). Both those parameters were correlated (r = 0.424; *p* < 0.001), and GDF-15 levels also correlated with other biomarkers of COVID-19 severity/mortality, namely, IL-6, CRP, ferritin, D-dimer and neutrophils, and inversely with lymphocyte count (Figure 1C). Additionally, those biomarkers also correlated with each other: IL-6 with CRP, D-dimer and neutrophils, CRP with D-Dimer and neutrophils, ferritin with D-dimer. Lymphocyte count also inversely correlated with IL-6, CRP, D-dimer and neutrophils (data not shown). As expected, an association was observed between GDF-15 levels and age in both the COVID-19 and healthy control individuals (data not shown).

**Figure 2:**
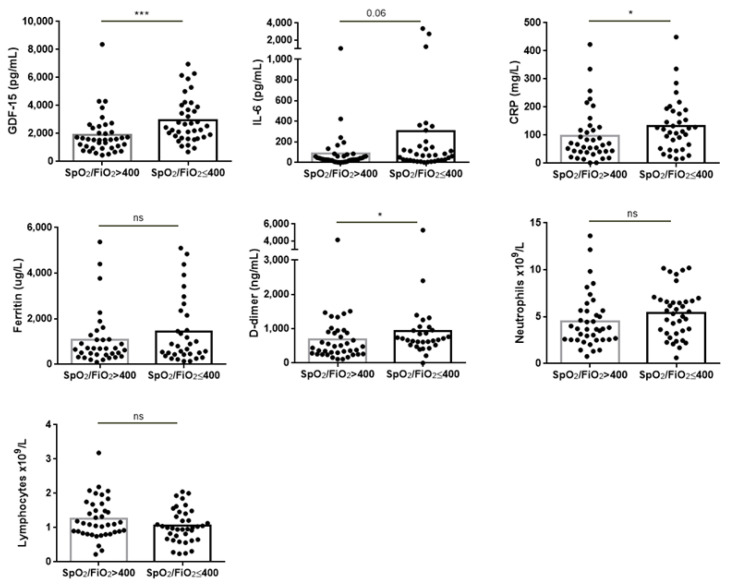
Analytical parameters in patients with COVID-19 segregated according to respiratory function (SpO_2_/FiO_2_). Comparison of GDF-15, IL-6, CRP, ferritin, D-dimer, neutrophils and lymphocyte counts in patients with SpO_2_/FiO_2_ > 400 (n = 39) and SpO_2_/FiO_2_ ≤ 400 (n = 38). The Mann–Whitney test was used for statistical analysis. *** *p* < 0.001; * *p* < 0.05; ns: non-significant.

When patients were segregated according to respiratory function, those with low SpO_2_/FiO_2_ values (≤400) were observed to have higher levels of GDF-15, CRP and D-dimer, and also tended to have higher IL-6 levels, while no differences were observed for ferritin, neutrophils or lymphocytes (Figure 2).

GDF-15 was inversely correlated with both SpO_2_ (r = 0.408; *p* < 0.001) and SpO_2_/FiO_2_ (r = 0.493; *p* < 0.001). No differences in demographics or comorbidities were observed between groups segregated according to SpO_2_/FiO_2_ ratios (data not shown). ROC analyses demonstrated that GDF-15 ≥ 1675 pg/mL best identified poorer respiratory function (SpO_2_/FiO_2_ ≤ 400) (Table 2).

## 4. Discussion

Our results showed that serum GDF-15 levels were increased in patients with COVID-19 and also correlated with other biomarkers of severity. Additionally, of all the biomarkers analysed, in AUC terms, GDF-15 levels best identified patients with poorer respiratory function.

It is well established that GDF-15 may rise substantially in the elderly [12]. In both healthy control and the COVID-19 cohort we found a significant correlation between GDF-15 levels and age, however, both cohorts did not differ significantly in age nor in sex, so the increased levels of GDF-15 in COVID-19 patients were not influenced by a demographic bias.

To our knowledge, this is the first study that associates GDF-15 levels with SpO_2_/FiO_2_ ratios, typically used to monitor pulmonary ventilation and lung injury. Our results show that GDF-15 levels were higher in patients with lower SpO_2_/FiO_2_ ratios reflecting pulmonary impairment. Supporting our finding is a previous study that has correlated GDF-15 with hypoxemia [9].

Previous studies have shown that elevated levels of proinflammatory cytokines, such as IFN-γ, TNF-α, IL-6 and IL-8, are associated with severe lung injury and adverse outcomes, suggesting that the magnitude of cytokine storm is associated with COVID-19 severity. Although inflammatory biomarkers such as CPR and D-dimer were also higher in patients with lower SpO_2_/FiO_2_ ratios, the AUC for GDF-15 levels better reflected patients with a poorer respiratory condition, so GDF-15 is a more accurate biomarker to detect those patients than CPR or D-dimer.

It is important to highlight the potential value of GDF-15 as a prognostic factor for COVID-19. Since the primary cause of COVID-19 mortality is ARDS [13], early detection of patients with a high probability of developing ARDS is crucial for their optimal management. Increased GDF-15 levels have been associated with a higher risk of both intensive care unit admission [9] and mortality [9,10]. Our finding that GDF-15 levels are higher on admission in longer-stay patients hospitalized with COVID-19 supports a potential role for quantifying GDF-15 as a means of promptly evaluating prognosis in these patients.

Our study has two main limitations. First, we could not establish an association between GDF-15 levels and mortality due to the low mortality in our cohort, and second, we did not take serial measurements in order to analyse the possible role of GDF-15 in COVID-19 follow-up.

In conclusion, GDF-15 has a potential role in stratifying patients with COVID-19 by severity. Our data support the value of GDF-15 as a biomarker associated with pulmonary impairment in COVID-19.

## Figures and Tables

**Table 1:** Demographic, clinical and laboratory data.

	Healthy Controls (n = 20)	COVID-19 Patients (n = 84)
Female % (n)	60 (12)	40.5 (34)
Age, years (mean±SD)	58.6 (±13.9)	55 (±11.8)
Previous pulmonary pathology		
Asthma % (n)		2.38 (2)
COPD % (n)		2.38 (2)
Pulmonary malignancy % (n)		1.19 (1)
Non-specific interstitial pneumonia % (n)		1.19 (1)
Other comorbidities		
Smoker % (n)		1.2 (1)
Ex-smoker % (n)		19 (16)
SAD % (n)		8.3 (7)
DM % (n)		11.9 (10)
HT % (n)		34.5 (29)
DLP % (n)		27.4 (23)
Laboratory parameters		
IL-6 (pg/mL)		47.27 (23.61–119.1)
CRP (mg/L)		92.95 (44.1–145.2)
Ferritin (ug/L)		695.5 (405–1526)
D-dimer (ng/mL)		623 (359–958)
Neutrophil count (×10^9^/L)		4.36 (2.70–6.46)
Lymphocyte count (×10^9^/L)		1.04 (0.81–1.45)
Ventilation		
Air % (n)		35.71 (30)
Oxygen requirement % (n)		60.71 (51)
Invasive ventilation % (n)		3.57 (3)

COPD, chronic obstructive pulmonary disease; SAD, systemic autoimmune disease; DM, diabetes mellitus; HT, arterial hypertension; DLP, dyslipidaemia; IL-6, interleukin-6; CRP, C-reactive protein.

**Table 2:** AUC analysis of inflammatory biomarkers in patients with COVID-19.

	ROC AUC (95% CI)	*p*-Value
GDF-15	0.729 (0.602–0.857)	0.002
IL-6	0.615 (0.466–0.763)	0.126
CRP	0.642 (0.498–0.786)	0.580
Ferritin	0.590 (0.444–0.736)	0.230
D-dimer	0.671 (0.535–0.807)	0.230

AUC, area under the receiver operating characteristics (ROC) curve; GDF-15, growth differentiation factor 15; IL-6, interleukin-6; CRP, C-reactive protein.

## Data Availability

The data presented in this study are available on request from the corresponding author. The data are not publicly available due to ethical restriction.
